# HELICAL COMPUTERIZED TOMOGRAPHY CAN MEASURE SUBCUTANEOUS, VISCERAL AND TOTAL FAT AREAS?

**DOI:** 10.1590/0102-672020210003e1591

**Published:** 2022-01-05

**Authors:** Guilherme WENDLER, Paulo Afonso Nunes NASSIF, Osvaldo MALAFAIA, Eduardo WENDLER, Ilana Barrichello Torres WENDLER, Luiza Marcelli CIRPIANI

**Affiliations:** 1 Postgraduate Program in Principles of Surgery, Mackenzie Evangelical Faculty of Paraná/Medical Research Institute, Curitiba, PR, Brazil; 2 Rocio Hospital, Campo Largo, PR, Brazil

**Keywords:** Computed tomography, Obesity, Visceral fat, Subcutaneous fat, Total fat, Tomografia computadorizada, Obesidade, Gordura visceral, Gordura subcutânea, Gordura total

## Abstract

***Background*:**

Abdominal obesity or android obesity, that is, the increase in adipose tissue in the abdominal region, is considered a risk factor for several morbidities. Different ways of quantifying it have been proposed, one method is the measurement of the abdominal fat area by computed tomography.

***Aim*:**

To establish correspondence between the groups defined by degree of obesity in relation to the total, subcutaneous and visceral fat area.

***Methods*:**

Cross-sectional observational study carried out through the analysis of tomographic examinations. Horos v3.3.5 medical image visualization software was used, with abdominal tomography in a single cut including the L4 vertebral body and the umbilical scar, to obtain the areas of total, visceral and subcutaneous fat.

***Results*:**

Of the 40 patients, 10 had grade II obesity, 23 grade III and 7 superobese. The amount of total fat showed an increase in relation to the degree of obesity. Visceral fat did not show significant differences between the degrees of obesity, but the data showed a lower average in the group of obesity grade II. The area of subcutaneous fat, as well as total fat, showed an increase in its measurements, according to the progression of the patients’ BMI, but there was no statistical significance in this difference between the groups of grade II and super-obese individuals.

***Conclusion*:**

The area of total and subcutaneous fat showed an increase in its measurements according to the progression of the BMI groups, which did not happen with visceral fat.

## RESUMO

***Racional*:**

A obesidade abdominal ou a obesidade androide, isto é, o aumento de tecido adiposo na região abdominal, é considerada fator de risco para diversas morbidades. Diferentes formas de quantificá-la foram propostas, sendo um dos métodos a medida da área da gordura abdominal pela tomografia computadorizada.

***Objetivo*:**

Estabelecer correspondência entre os grupos definidos por grau de obesidade em relação a área de gordura total, subcutânea e visceral.

Estudo observacional analítico transversal realizado através da análise de exames tomográficos. Utilizou-se software de visualização de imagens médicas Horos v3.3.5, com tomografia abdominal em um único corte incluindo corpo vertebral de L4 e a cicatriz umbilical, para obter as áreas de gordura total, visceral e subcutânea.

***Resultados*:**

Dos 40 pacientes 10 apresentavam obesidade grau II, 23 grau III e 7 superobesos. A quantidade de gordura total demonstrou aumento com relação ao grau de obesidade. A gordura visceral não apresentou diferenças significativas entre os graus de obesidade, porém os dados demonstraram média menor no grupo de obesidade grau II. A área de gordura subcutânea, assim como a gordura total, apresentou aumento de suas medidas, conforme a progressão do IMC dos pacientes, porém não houve significância estatística nesta diferença entre os grupos de obesos grau II e superobesos.

***Conclusão*:**

A área de gordura total e subcutânea apresentaram aumento de suas medidas conforme a progressão dos grupos de IMC, o que não aconteceu com a gordura visceral.

## INTRODUCTION

The prevalence of obesity has increased in the last decade and several prospective cohort studies have shown that obese people, defined by body mass index (BMI), are at increased risk of arterial hypertension and cardiovascular disease as well as of mortality from these causes^18^ ^,^ ^23^. In addition, the distribution of fat deposits, such as visceral abdominal adipose tissue (VAT), regardless of general obesity, has been associated with cardiometabolic risks^11^ and others^5^.

Abdominal obesity is composed of subcutaneous and visceral fat, the latter presenting metabolic and functional characteristics that distinguish it from that located in other anatomical regions, representing a greater predictive value for cardiovascular disease^21^.

In view of the relevance of visceral fat in the study of metabolic syndrome, several methods have been proposed to assess the distribution of body fat and quantify intra-abdominal adiposity. There are a variety of techniques for the assessment of body composition, such as anthropometric measurements (waist circumference, waist / hip ratio, waist-to-height ratio, taper index, and sagittal diameter) and image measurements (computed tomography, magnetic resonance and ultrasound)^1^ ^,^ ^6^.

In the last few decades, it has been found that adipocytes are responsible for the secretion of various types of hormones and cytokines. It is known that leptin, produced and secreted mainly in adipocytes, is involved in the neuroendocrine regulation of adiposity and its metabolic sequelae. Plasma concentrations have been reported to be associated with BMI, fat percentage and total body fat mass assessed by dual energy X-ray absorptiometry. However, there is still controversy about which fat mass is strongly associated with the plasma leptin level, that is, whether it is visceral abdominal adipose tissue (VAT) or subcutaneous abdominal adipose tissue (SAT)^22^.

Individuals with fat accumulation in the upper body are more susceptible to the progression of atherosclerosis than those with less. In the 1980s, the method of assessing the VAT area by computed tomography was reported, and it was suggested that this fat plays an important role in the development of diabetes mellitus, systemic arterial hypertension and hyperlipidemia. However, it remains to clarify how and to what extent individual adipose tissue contributes to abdominal metabolism, that is, the direct relationship that each type of fat may have with the degree of obesity^19^.

Helical computed tomography (CT) is a mode of volume acquisition by X-rays. Helical data are obtained by moving the table with the patient at constant speed, while the X-ray tube rotates continuously with sustained exposure9.

Helical CT is known to have several important potential advantages for examining the abdomen. In comparison with the standard type, it is less sensitive to movement artifacts and, due to the acquisition of volume during respiratory suspension, there is no incorrect and different record in respiratory movements^9^.

Eastwood et al., (2013)^2^ presented internal software to measure the muscle and fat area in axial tomography and compare it with several quantification methods. The software analyzes body composition on computed tomography and has proved to be reliable in the quantification of fat and muscle tissue. Fox, et al. (2007)^4^ studied the compartments of VAT by CT that may confer a higher metabolic risk. The usefulness of measuring VAT and SAT in association with metabolic risk factors has not been well described in a population-based scenario. Among women and men, SAT and VAT were significantly associated with several changes (blood pressure, fasting glucose, lipidogram, hypertension, diabetes mellitus) and mainly with the metabolic syndrome.

CT is an advanced and fairly new technique used to diagnose various disorders. However, its application in the assessment of the distribution of body fat mass is recent. Using helical CT it was shown that the percentage of visceral and subcutaneous fat was significantly higher in women than intra-abdominal fat in men^15^.

The accumulation of adipose tissue in the abdominal region is considered a risk factor for several morbidities and, given the relevance of visceral fat in the study of the metabolic syndrome, several methods have been proposed to assess the distribution of body fat and quantify intra-abdominal adiposity^12^. This aspect justifies the performance of this work, as there are not many studies that used CT as a form of quantitative assessment of abdominal fat and data in the literature that correlate these findings with the degree of obesity.

Thus, the objective of this research was to establish a correspondence between the groups clinically defined by degree of obesity and their quantifications of the areas of total fat, subcutaneous and visceral fat defined by CT.

## METHOD

This is an observational analytical cross-sectional study carried out at Hospital do Rocio, Campo Largo, PR, Brazil, through the analysis of tomographic findings correlated with the clinical data prospectively obtained from patients who are candidates for surgical treatment of obesity. It was approved by the Research Ethics Committeê of Faculdade Evangélica Mackenzie do Paraná́ - CEP / FEMPAR, according to the attributions defined in Resolution 466/12 CNS under opinion number 1,836,670. The technical standards used in this work followed the guidelines determined by the Mackenzie Standards of 2019.

Forty obese patients recruited for Roux-en-Y gastric bypass were recruited from August 2018 to July 2019.

Inclusion criteria were patients who agreed to participate in the study by signing the informed consent form; eligible for the proposed operation and who had a BMI> 35 kg/m^2^ associated with diabetes mellitus, systemic arterial hypertension or a BMI> 40 kg/m^2^. Exclusion criteria were patients younger than 18 and older than 60 years; weight greater than 150 kg (weight limit of the movable table) and with incomplete anamnesis form.

### Data collect

BMI was calculated using the formula weight divided by height at the table in meters determining the degree of obesity (Tables 1 and 2).

**TABLE 1:** Descriptive statistics of demographic variables and comorbidities

VariABLE	Classif	Results*
Age |(years)		37,5±9,5 (20-57)
	20-29	8 (20)
	30-39	15 (37,5)
	40-49	11 (27,5)
	50-59	6 (15)
Gender	Male	6 (15)
	Female	34 (85)
BMI		43,7±4,9 (35,8-56)
	Level II	10 (25)
	Level III	23 (57,5)
	Superobesity	7 (17,5)
Total fat (cm^2^)		808±153 (493-1191)
Visceral (cm^2^)		196±60 (73-298)
Subcutaneus (cm^2^)		612±162 (195-1044)

* Described by mean ± standard deviation (minimum - maximum) or by frequency (percentage)

**TABLE 2:** Homogeneity between groups

Variable	Classif	Obesity (BMI)			p*
		Level II (n=10)	Level III (n=23)	Superobesity (n=7)	
Age		38 ± 8 (24 - 51)	37 ± 10 (20 - 57)	39 ± 11 (25 - 53)	0,896
Gender	Male	2 (20)	2 (8,7)	2 (28,6)	
	Fem	8 (80)	21 (91,3)	5 (71,4)	0,382

*ANOVA com um fator ou teste de Qui-quadrado, p<0,05

All acquisitions were made using the GE Optima CT520 Series 16-channel helical computed tomography machine using parameters of 180 mA, 120 KV, slice thickness of 5 mm and step 1.75:1 (table speed in relation to slice thickness)

All patients underwent CT in a single series of axial slices, with a matrix of 512x512 pixels, in the supine position, without the injection of intravenous contrast, which included images from the upper hepatic border to the pelvis.

### Calculation of body fat mass

The measurement of body fat mass was performed using the iMAC hardware, using the macOS 10.14.1 operating system and the Horos v3.3.5 medical image visualization software (https://horosproject.org/).

**FIGURE 1:**
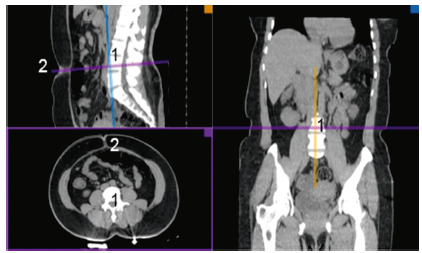
Standardization of the cut level on the CT to quantify the fat.

Multiplanar reconstruction of the axial sections was performed, being considered a single cut suitable for the evaluation that should include the L4 vertebral body of the lumbar spine and the umbilical scar at its origin in the abdominal wall (Figure 1).

1=corpo vertebral (L4); 2=cicatriz umbilical

The Thresholding image tool was used, which is a method for segmenting the image using the gray scale. The thresholds used to highlight the pixels form a defined area. The defined threshold was fat.

Considering the Hounsfield scale - tomography measurement unit - the range from -190 to -30 UH was used as fat densit^2^.

**FIGURE 2:**
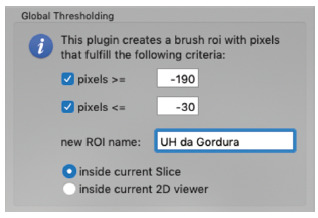
Thresholding tool with fat thresholds

After delimiting the pixels, the area per cm^2^ that corresponds to that of total abdominal fat was obtained.

**FIGURE 3:**
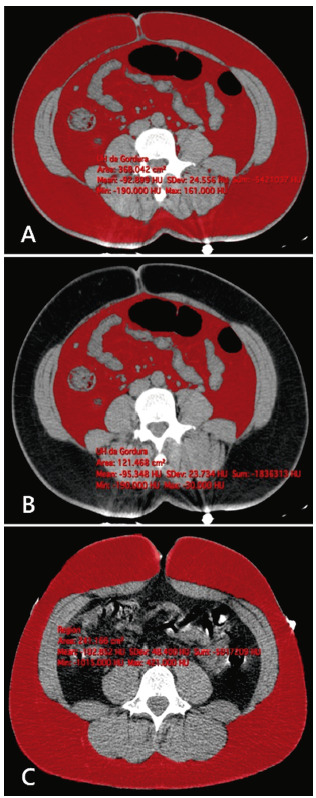
Examples of measurement of the fat area: A) total abdominal; B) visceral abdomen; C) subcutaneous.

Red = total abdominal fat; white = spine; gray = muscles; black / gray = intestine

The SAT was manually removed and as a consequence, the VAT area in cm^2^ was obtained, which is the parameter used as a correlation measure. Consequently, to calculate the SAT area, subtract the area from the total fat area value.

### Statistical analysis

The results of quantitative variables were described by means, standard deviations, minimum and maximum values. Categorical variables were described by frequencies and percentages. The three groups defined by BMI (obesity grade II, obesity grade III and super obesity) were compared in relation to quantitative variables using the model of analysis of variance (ANOVA) and the model of analysis of covariance adjusted for diabetes. For multiple post-hoc comparisons, the Bonferroni test was used. Categorical variables were analyzed considering the chi-square test. To assess the correlation between two quantitative variables, Pearson’s correlation coefficients were estimated. The condition of normality for continuous variables was assessed by the Kolmogorov-Smirnov test. Values ​​of p <0.05 indicated statistical significance. The data were analyzed using the computer program Stata / SE v.14.1. StataCorpLP, USA

## RESULTS

The sample included 40 patients, after the exclusion criteria were applied. The mean age was 37.5 years, with an average BMI of 43.7 kg / m2. The measurements of areas (cm2) of total, visceral and subcutaneous fat were 808,196 and 612, respectively (Table 1).

### Homogeneity of groups

The patients were divided into groups based on the BMI calculation, resulting in 10 grade II obese, 23 grade III and 7 superobese. The groups presented variables (ages, gender, BMI) without significant differences, confirming the homogeneity between them (Table 2).

### Comparison of the groups defined by BMI in relation to the total fat variable

**FIGURE 4:**
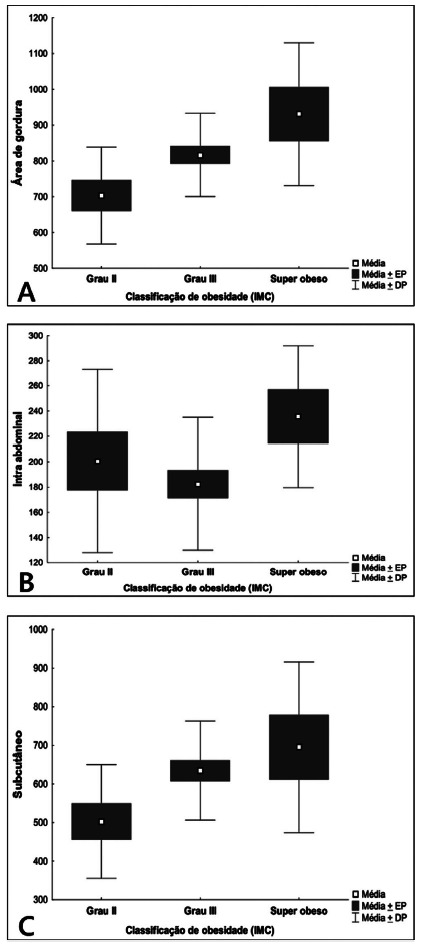
Area averages x groups: A) total fat; B) visceral; C) subcutaneous

The analysis of the amount of total fat showed an increase in relation to the degree of obesity. There was statistical significance of this difference when comparing the grade II groups to the superobese group (p = 0.006, Tables 3 and 4, Figure 4.

**TABLE 3:** Comparison of the groups defined by the BMI in relation to the variables area of fat, visceral and subcutaneous

Variable	Obesity Level	n	Avarage ± Standart desv (min - max)	p*	p**
Total fat (cm^2^)	LEVEL II	10	703±135 (493-846)	0,007	0,052
	LEVEL III	23	817±116 (656-1090)		
	Superobesity	7	931±199 (706-1191)		
Visceral (cm^2^)	LEVEL II	10	201±73 (73-298)	0,119	0,052
	LEVEL III	23	182±53 ( 85-278)		
	Superobesity	7	236±56 (147-293)		
Subcutanous (cm^2^)	LEVEL II	10	503±147 (195-654)	0,029	0,261
	LEVEL III	23	634±128 (407-912)		
	Superobesity	7	695±221 (468-1044)		

*ANOVA com fator p<0,05; **ANOVA incluindo DM como covariável p<0,05

**TABLE 4:** Comparison of the groups defined by the BMI in relation to the variables total fat área

Variable	Degrees Compared	p*
Total Fat (cm^2^)	LEVEL II x LEVEL III	0,109
	LEVEL II x superobesity	0,006
	LEVEL III x superobesity	0,188

*Teste post-hoc de Bonferroni, p<0,05

### Comparison of groups defined by BMI in relation to the visceral fat variable

The analysis of the amount of visceral fat did not show significant differences between the three groups; however, the data showed a lower average in the grade II group (Table 3 and Figure 4B)

### Comparison of the groups defined by BMI in relation to the subcutaneous fat variable

The area of subcutaneous fat, as well as total fat, showed an increase in its measurements, according to the progression of the BMI groups. However, the statistical significance of this difference was shown in the comparison between the grade II and superobese groups (Table 5 and Figure 4C).

**TABLE 5:** Comparison of the groups defined by the BMI in relation to the subcutaneous area variables

Variable	Degrees Compared	p*
Subcutaneous (cm^2^)	LEVEL II x LEVEL III	0,083
	LEVEL II x superobesity	0,043
	LEVEL III x superobesity	1

*Teste post-hoc de Bonferroni, p<0,05

## DISCUSSION

CT has been considered the most accurate and reproducible technique for measuring body fat, particularly abdominal adipose tissue, allowing the differentiation of subcutaneous and visceral adiposity in this region, being considered since 1990 as the “gold standard” method for determining the visceral fat^14^.

Magnetic resonance imaging is a safe imaging method, accurate for measuring visceral and subcutaneous adipose tissue. It is a favorable alternative and not exposed to radiation to measure abdominal fat content in clinical research^8^; however, it is less likely to be influenced by the respiratory artifact and most MRI systems have a diameter of 60 cm, limiting it to patients with higher degrees of obesity. CT and MRI are the two best methods for assessing body fat. CT is more available, has a more accessible cost and better accommodates more obese patients.

One of the great current advantages of measuring areas or volumes of intra-abdominal body fat by CT over measurement by conventional methods is to reflect the body fat mass more accurately^16^ ^,^ ^17^. However, in the study by Kobayash et al. (2002)^9^ this correspondence was not evaluated. In this work, analyzing the amount of total fat, there was an increase in relation to the degree of obesity, with statistical significance in this difference when comparing the grade II groups to the superweight group (p = 0.006), corroborating with Vohl (2004)^24^ who, differently from VAT to SAT, it has a higher correlation with degree of obesity.

Shuster et al. (2012)^20^ report that one of the findings that is little valued in CT exams is fat, specifically in this case, abdominal. It is believed that it is due to the lack of a level of evidence that justifies its citation in the radiological reports; however, in this study, a correspondence was established between the degree of obesity and the level of visceral, subcutaneous and total fat.

Kobayash et al. (2002)^9^ state that as the importance of these various discoveries becomes apparent, the requirement for improved techniques that can safely measure intra-abdominal fat mass has become equally evident; Shuster et al. (2012)^20^ believe that abdominal fat (visceral and subcutaneous) may be part of radiological reports, contributing positively to medical practice. The need for accurate and clinically convenient measures to quantify VAT is evident. However, it is also essential to develop quantitative criteria to define visceral obesity in relation to these metabolic disorders. This study demonstrated that by helical CT it may be possible to compare groups defined by BMI (degree of obesity) with areas of visceral, subcutaneous and total fat.

Here, the quantitative method was used to assess abdominal fat validated by Kobayash et al. (2002)^9^ which has good reproducibility, is fast and can be applied in other studies. They state that an area of ​​visceral fat of 130 cm^2^ in both men and women of various age groups is strongly related to metabolic disorders; however, in the present study the results showed an average visceral fat area of ​​196 cm^2^ in the three groups; however, the correlation of these groups with the metabolic syndrome has not been evaluated.

Most studies use the Hounsfield Unit with a range of -190 to -30 pixels for subcutaneous adipose tissue and VAT quantification in CT studies. There is no consistent threshold that can be applied to the segmentation of adipose tissue by resonance; however, there has been significant growth in the automation of the analysis process in recent years^3^. Also here the same unit was used with an interval of -190 to -30 pixels, obtaining the area in cm^2^ that corresponds to the total abdominal fat; the appropriate cut-off level for assessment should include the L4 vertebral body and the umbilical scar at its origin in the abdominal wall.

The regional distribution of fat has a good influence on health, even in the absence of generalized obesity^13^. Studies suggest that visceral or intra-abdominal fat is the most harmful due to its hormonal functions. The subcutaneous has the main functions of maintaining body fat, storing energy more efficiently than visceral fat and hormonal production^24^. This study compared the degree of obesity with visceral fat, demonstrating that it did not present significant differences between the three groups; however, the data showed a lower average in the grade II obesity group. Several researchers have attempted to quantify body composition using CT, and several methods have been developed over the years. Yoshizumi et al.^25^ developed a standardized technique for measuring fat using manual measurements of CT data, and showed that the manual measurements were almost identical to quantify the abdominal fat area^7,25.^ In this study, the Horos image visualization software was used, following the methodology of Kobayashi et al. (2002)^9^ that demonstrated a good result.

Subcutaneous fat has a significant correlation with BMI, but not with visceral fat. Thus, it is concluded that the BMI should not be used in isolation for the screening and evaluation of comorbidities^10^. There are other parameters for this analysis, such as the waist and height ratio.

The correlation between degrees of obesity, metabolic changes and quantification of different types of fat should be the subject of further studies. The growth in the fields of artificial intelligence may be a future direction to provide accurate and fully automated 3D segmentation of adipose tissue deposits.

## References

[1] Alvarez MM, Vieira AC, Sichieri R, Veiga GV (2008). Association between central body anthropometric measures and metabolic syndrome components in a probabilistic sample of adolescents from public schools. Arq Bras Endocrinol Metabol.

[2] Eastwood. S.v. (2013). Thigh fat and muscle each contribute to excess cardiometabolic risk in South Asians, independent of visceral adipose tissue. Obesity.

[3] Fang H, Berg E, Cheng X, Shen W (2018). How to best assess abdominal obesity. Curr Opin Clin Nutr Metab Care.

[4] Fox C.S. (2007). Abdominal visceral and subcutaneous adipose tissue compartments: association with metabolic risk factors in the Framingham Heart Study. Circulation.

[5] Freitas BA, Loth CAT, Swarowsky GL, LourenÇo GM, Fillmann LS, Fillmann HS, Santos ML, Padoin AV (2020). Are obesity and adenoma development associated as colorectal cancer precursors. Arq Bras Cir Dig.

[6] Hirooka M (2005). A technique for the measurement of visceral fat by ultrasonography comparison of measurements by ultrasonography and computed tomography. Intern Med, Tokyo,.

[7] KIM S. S. (2019). Semiautomatic software for measurement of abdominal muscle and adipose areas using computed tomography: A STROBE-compliant article. Medicine (Baltimore).

[8] Klopfenstein B.J. (2012). Comparison of 3 T MRI and CT for the measurement of visceral and subcutaneous adipose tissue in humans. The British Journal of Radiology.

[9] Kobayashi J. (2002). A novel method of measuring intra-abdominal fat volume using helical computed tomography. International Journal of Obesity.

[10] Lima W.C. (2010). Análise da relação entre a estatura e o perímetro abdominal em indivíduos portadores de percentuais normais de gordura. ABCD, arq. bras. cir. dig.

[11] Pak K. (2016). Comparison of Visceral Fat Measures with Cardiometabolic Risk Factors in Healthy Adults. PLoS ONE.

[12] Petribú MMV (2012). Métodos de avaliação da gordura abdominal. Rev Bras Nutr Clin.

[13] Pitanga F.J.G., Lessa I (2005). Indicadores antropométricos de obesidade como instrumento de triagem para risco coronariano elevado em adultos na cidade de Salvador - Bahia. Arquivos Brasileiros De Cardiologia.

[14] Ribeiro FF (2006). Gordura visceral e síndrome metabólica mais que uma simples associação. Arq Bras Endocrinol Metab.

[15] Rogalla P (1998). Low-dose spiral computed tomography for measuring abdominal fat volume and distribution in a clinical setting. Eur J Clin Nutr.

[16] Ross R (1993). Adipose tissue distribution measured by magnetic resonance imaging in obese women. Am J Clin Nutr.

[17] ross R (1992). Quantification of adiopose tissue by MRI: relationship with anthropometric vari- ables. J Appl Physiol.

[18] Santoro S, Aquino CGG, Mota FC, Artoni RF (2020). Does evolutionary biology help the understanding of metabolic surgery A focused review. Arq Bras Cir Dig.

[19] Seidell J.C. (1988). Abdominal fat depots measured with computed tomography: effects of degree of obesity, sex, and age. European Journal Clinical Nutrology.

[20] Shuster A. (2012). The clinical importance of visceral adiposity: a critical review of methods for visceral adipose tissue analysis. The British Journal of Radiology.

[21] SILVA JLT (2006). Distribuição centrípeta da gordura corporal, sobrepeso e aptidão cardiorres- piratória associação com sensibilidade insulínica e alterações metabólicas. Arq Bras Endocrinol Metab.

[22] Tahara N. (2015). Clinical and Biochemical Factors Associated With Area and Metabolic Activity in the Visceral and Subcutaneous Adipose Tissues by FDG-PET/CT. The Journal of Clinical Endocrinology & Metabolism.

[23] Vargas JA, Bonato RCS, Orenha ES, Sales-Peres SHC (2020). Assessment of alveolar bone pattern in obese and non-obese women, before and after bariatric surgery a prospective cohort study. Arq Bras Cir Dig.

[24] Vohl MC (2004). A survey of genes differentially expressed in subcutaneous and visceral adipose tissue in men. Obesity Research.

[25] Yoshizumi T (1999). Abdominal fat standardized technique for measurement at CT. Radiology.

